# Administration of probiotics to healthy volunteers: effects on reactivity of intestinal mucosa and systemic leukocytes

**DOI:** 10.1186/s12876-022-02185-1

**Published:** 2022-03-05

**Authors:** Christina Stene, Andrada Röme, Ingrid Palmquist, Caroline Linninge, Göran Molin, Siv Ahrné, Louis Banka Johnson, Bengt Jeppsson

**Affiliations:** 1 Department of Surgery, Skåne University Hospital/Malmö, Lund University, 205 02 Malmö, Sweden; 2 Food Hygiene, Department of Food Technology, Engineering and Nutrition, Lund University, 221 00 Lund, Sweden

**Keywords:** Probiotics, Bifidobacteria, Lactobacilli, Mucosal inflammation

## Abstract

**Background:**

Oral administration of health-promoting bacteria is increasingly used in clinical practise. These bacteria have anti-inflammatory characteristics and modulate the immune system without major reported side effects. The mechanisms of action are not yet fully defined. Our aim was to study systemic effects of probiotics by measurements of leukocytes as well as local effects on rectal mucosal biopsies after adding a standardized inflammatory stimulus *in vitro*.

**Methods:**

Fourteen healthy subjects were randomized to receive 10^10^ colony forming units/day orally of the probiotic strain *Lactiplantibacillus plantarum* 299 (Lp299), n = 7, or *Bifidobacterium infantis* CURE21 (CURE21), n = 7, for six weeks. Rectal biopsies were taken before and after ingestion of either probiotic strain product, for stimulation *in vitro* with tumour necrosis factor alpha (TNF-α) at 10 and 100 ng/ml respectively up to 8 h. Blood tests were sampled before and after treatment. Lactate dehydrogenase (LDH) confirmed viable tissue.

**Results:**

Composition of the intestinal microbiota was not changed. Systemic leukocytes decreased after administration of CURE21 (*P*<0.05) and Lp299 (*P*<0.01). Levels of the pro-inflammatory cytokine IL-6 in rectal mucosa after stimulation with TNF-α were attenuated after ingestion of Lp299. No effect was seen with CURE21.

**Conclusions:**

Administration of these probiotic strains to healthy humans show both a systemic and local reduction of inflammatory response by lowering leukocyte counts, and for Lp299 IL-6 levels in rectal mucosa. Probiotics may play an important role in the reduction of inflammatory responses expected after trauma during surgery or after pelvic irradiation.

*Trial registration* Clinical Trials, registration number NCT01534572, retrospectively registered (http://www.clinicaltrials.gov).

## Background

The human body contains a rich endogenous bacterial microbiota that is going through different phases of dynamic functional activity during one’s lifetime. Some gut bacteria have been shown to enhance health, whilst others may be harmful to it [1]. The healthy subject is in a balanced relationship with its microbial community. When dysbiosis occurs, the imbalance may lead to or enhance an existing disease condition.

Critical functions of the resident microbiota include protection against epithelial cell injury [2], regulation of host fat storage [3], stimulation of intestinal angiogenesis [4] and development of normal mucosal immunity [5]. In recent years multiple studies have shown beneficial effects of enteral administration of non-pathogenic bacteria in cases with sepsis or severe trauma with increased inflammation and gut barrier dysfunction where overgrowth of potentially pathogenic bacteria is observed. Mechanisms of action have not been fully clarified but it is postulated that there is a restoration of deranged barrier function, a counterbalance of pathogenic bacteria and a stimulation of the immune function by the presence of non-pathogenic bacteria [6]. The most studied probiotics have been chosen from the genus *Bifidobacterium* and the family *Lactobacillaceae*.

Adult faecal bacteria may comprise of up to 25% *Bifidobacterium* and in infants even a higher percentage of about 80%. Bifidobacteria as a probiotic mediator has been widely studied in gastrointestinal disorders in humans and animals, for example during intestinal infections, colonic transit disorders as well as colonic adenoma and colon cancer. *B. infantis* CURE21, (CURE = Colonic Ultima Ratio Efflux), (CURE21), has earlier been isolated from the rectum of small children (G. Molin, pers comm). CURE21 has been proven to show anti-inflammatory capacity in a rat model [7], where *B. infantis* strains with and without a combination of oligofructose and inulin (OFI) attenuated inflammation in DSS-induced colitis in rats.

Since the era of Metchnikoff, lactobacilli have been shown to be associated with the human gastrointestinal tract. Lactobacilli ferments carbohydrates to lactic acid and is linked to mucous membranes of the oral cavity, gastrointestinal tract (GIT) and vagina in human beings and animals. Their special property of resistance to acidity allows several taxa of lactobacilli to grow in spite of low pH. *Lactiplantibacillus plantarum* (former called *Lactobacillus plantarum*) [8], (Lp), has been isolated from the intestinal mucosa of a specific healthy subject and has shown a pronounced ability to attach to human mucosal cells *in vitro*, where its adhesion is dependent on a mannose-binding adherence mechanism [9, 10]. *L. plantarum* 299 (Lp299) has been demonstrated to exert beneficial effects in the GIT acting to prevent pathological permeability of the intestine [11].

Whilst most studies have measured the effect of *in vitro* exposure of epithelial cells to different probiotic bacteria we think the effects of these bacteria cannot rightly be observed only in a static medium but rather postulate that they exert their influence in a complex medium of varied bacterial composition and any assessment of these functions has to be carried out during a longer period of time. Probiotics probably work by adapting the gastrointestinal tract mucosa to the present constellation of varied bacterial flora. We wanted to investigate if intestinal mucosa could be strengthened and be prepared to withstand a later injury as such situations are common in clinical practice. We have thus chosen to investigate the effect of ingestion of two commercially available bacterial probiotic strains (*L. plantarum* 299 and *B. infantis* CURE21) on intestinal mucosa *in vivo* for a longer period. We study both local and systemic effects of the abovementioned probiotics on the gut mucosa after an ingestion period of six weeks. Immune parameters were measured systemically and locally by exposing mucosal biopsies from the rectum to a standardized inflammatory stimulus by the potent pro-inflammatory cytokine tumour necrosis factor α (TNF-α) which is known to play a fundamental role in the pathogenesis of impaired mucosal function [12–14].

## Methods

Fourteen healthy volunteers without symptoms from the gastrointestinal tract were recruited by open invitation and passed all formal procedures. The trial was approved by the Human Ethics Committee of Lund University, Sweden (LU738-03 2003-12-15, Dnr 538/2005 2005-11-02), as well as registered with Clinical Trials 2012-02-16, registration number NCT01534572 (http://www.clinicaltrials.gov). All and related trials for this intervention are registered. Written consent was obtained from all volunteers. The authors hereby attest that all volunteers included in the study gave both a verbal and signed informed consent to participate in the study. Study subjects were allocated by a procedure of randomly chosen envelopes to either *L. plantarum* 299 [DSM 6595] (batch number 11/4-05) or *B. infantis* CURE21 [DSM 15159] (batch number 12L05:5) ingestion, with seven persons in each group. During an eight-week period volunteers were advised to stop consumption of any probiotic products, traditional lactic acid fermented milk products and other lactic acid fermented products (e.g. brined olives, sauerkraut and pickled gherkins). Two weeks after abstaining from all fermented products blood and faecal samples were collected and thereafter the volunteers underwent a rigid recto-sigmoidoscopy where six biopsies were taken 15 centimeters up in the rectum using a two-millimeter biopsy forceps. All biopsies were weighed. After this visit volunteers started to ingest probiotics in freeze-dried powder form, dissolved in water, twice a day (10^10^ colony forming units/day), for six weeks. The administration of each probiotic was blinded to the volunteers and all the investigators except to the research nurse. No gut enema was used before biopsies.

After six weeks of probiotic administration a new set of samples was taken from blood and faeces as well as from the rectum by rigid recto-sigmoidoscopy, as done previously. Thus, the study subjects served as their own controls. Further, the subjects recorded daily frequency of defecation, stool consistency, flatulence activity, abdominal pain and also a subjective estimation of gastrointestinal function on a scale from 1 (best possible) to 10 (worst possible) in a study diary. Any altered habits were recorded with regards to physical activity, food, tobacco, alcohol and coffee/tea intake.

Faecal specimens were cultured for Lp299 and CURE21. Profiling of the gut microbiota was done by Terminal Restriction Fragment Length Polymorphism (T-RFLP) on rectal mucosal specimens. Separate quantitative PCR (qPCR) assays were used to estimate the presence of bacterial 16S rRNA genes of specified taxa, as described by Karlsson [15, 16].

A standardized inflammatory injury was created by *in vitro* stimulation of rectal biopsies with TNF-α for detection of the pro-inflammatory cytokine IL-6 and the anti-inflammatory cytokine IL-10 as described in the *in vitro* stimulation section.

Lactate dehydrogenase (LDH) was used as marker for mucosal viability, measured according to clinical routines at the laboratory, at 4 and 8 h in supernatant and tissue. Normal values being rated as less than 3.5 µkat/l. The ratio of LDH activity in the supernatant over total activity in tissue was calculated and used to estimate tissue viability.

### Blood tests

Blood samples were collected according to hospital routines and analysed for CRP (C-reactive protein), cytokines and fibrinogen. FACS (Fluorescence Activated Cell Sorting) analyses were done for leukocytes, lymphocytes, CD3, CD4, CD8, CD19, CD25-CD4, CD4/CD8, CD16+56, HLA-DR/CD3. Analyses were performed on EPICS^®^ XL-MCL^™^ Beckman Coulter^®^ 100161261 (Beckman Coulter^®^, Inc., CA, USA) for leukocytes and lymphocytes. CD4 were gated on CD45 positive lymphocytes, CD25 were gated on CD3/CD4 positive lymphocytes.

### Histology

One mucosal biopsy per subject was placed in formalin for 24 h and then embedded in paraffin, according to standard routines. The histological examination was performed by a blinded observer.

### Microbial analysis of the intestinal microbiota

For bacterial mapping of the mucosa, another biopsy per subject was weighed and then directly frozen in liquid nitrogen. DNA was extracted in BioRobot EZ1 (EZ1 DNA tissue kit and card, Qiagen, Hilden, Germany) according to Karlsson et al. [17] but 190 µl buffer G2 and 15 µl Proteinas K (Qiagen, Hilden, Germany) were used and samples were lysed for 2.5 h and shaken with 8-10 glass beads for 30 min before extraction in the robot.

The T-RFLP analysis was performed as described above. However, only restriction endonuclease *Msp*I was used for digestion of PCR products and threshold for terminal restriction fragments was set to 100 fluorescence units. Diversity indices (Shannon index and Simpson index) were calculated as previously described [16, 17] and the individual difference in diversity before and after treatment was compared between treatment groups.

Quantitative PCR (qPCR) was performed according to Karlsson [15, 18]. *Akkermansia muciniphilia-*like bacteria was quantified using primers AM1-F and AM2-R [19]. The detection limit was 10^4^ 16S rRNA copies per reaction for the *Enterobacteriaceae* assay and 10^2^ copies per reaction for all the other assays. The bacterial count is given as number of 16S rRNA genes per gram rectal mucosa.

Faecal samples were directly kept <8° C and analysed within 24 h for viable count of lactobacilli (Rogosa agar plates [Oxoid, Unipath LTD, Basingstoke, Hampshire, England] anaerobically incubated for 3 days in 37° C), *Enterobacteriaceae* (VRBG agar plates [Oxoid] incubated aerobically for 24 h in 37° C) and total plate count (RCM agar [Oxoid] incubated anaerobically for 3 days in 37° C). Typical colonies were picked from the Rogosa agar (Lp299) and RCM agar (CURE21) and typed with Randomly Amplified Polymorphic DNA (RAPD) according to the protocol of Johansson for tentative identification of Lp299 and CURE21 [20].

### *In vitro* stimulation

Three biopsies per individual were used for immune characterization. These biopsies were divided into halves, weighed and transported in medium (500 ml DMEM [Dulbecco’s Modified Eagle’s Medium, Sigma D5671], 5 ml L-Glutamine [Gibco, Invitrogen 25030-024], 5 ml PEST, 10,000 U Penicillin/10,000 µg Streptomycin/ml [Gibco, Invitrogen 15140-122, Fungizone^®^ 50 mg, Bristol-Myers Squibb, 0.5 mg/ml]) to the laboratory.

To conduct the *in vitro* stimulation the biopsies were placed in culture medium (500 ml DMEM [Dulbecco’s Modified Eagle’s Medium, Sigma D5671], 5 ml L-Glutamine [Gibco, Invitrogen 25030-024], 5 ml PEST, 10,000 U Penicillin/10,000 µg Streptomycin/ml [Gibco, Invitrogen 15140-122], 10% FCS [Integro, Saveen Werner FCS]) in culture wells (Costar^®^ Corning Incorporated, Corning, NY) and stimulated with TNF-α (R & D Systems Europe Ltd, Abingdon, Oxon, UK) in two different concentrations: 10 and 100 ng/ml. Treated samples were incubated at 37° C for 0 h, 4 and 8 h. A sample from the supernatant was withdrawn for cytokine analysis. New samples were drawn 4 and 8 h later. Concentrations and time points were chosen according to previous studies in animals performed by our group [21]. After stimulation with TNF-α, the medium was stored at -20° C before analysis of IL-6 and IL-10 using a specific Quantikine ELISA kit (R & D Systems, Oxon, UK).

### Statistical analysis

Statistical evaluations were performed with paired *t* test and Wilcoxon rank sum test, as well as Wilcoxon signed rank test, when appropriate. The results are presented according to the ingested bacteria for each group of volunteers, before and after the administration of probiotics, as median values and ranges. Differences were considered to be significant at *P* < 0.05. Statistical analyses of qPCR were performed using Mann-Whitney Rank Sum Test (Sigma Stat 3.0).

## Results

All healthy volunteers completed the experimental period, and no side effects of the probiotics were recorded.

### Demography

The number of healthy volunteers was 14 in total, nine women (64%) and five men (36%), with seven persons in each group. There was an equal distribution of age, gender and BMI between the two groups. The overall median age was 46 years (range 28–79) and the median BMI was 22 kg/m^2^ (range 19–27), (Table 1).

**Table 1:** Demographics

	n	Age median	Range	BMI median	range
Total	14	46.0	28–79	22.0	19–27
Women	9	48.0	28–79	21.0	19–27
Men	5	46.0	29–76	23.0	21–24

### Study diary

The volunteers did not experience any untoward effect of gut function before and after administration of probiotics. The number of defecations was reduced by 45% in the Lp299-group. No other changes were recorded.

### Blood tests

#### Leukocytes and lymphocytes

**Fig. 1:**
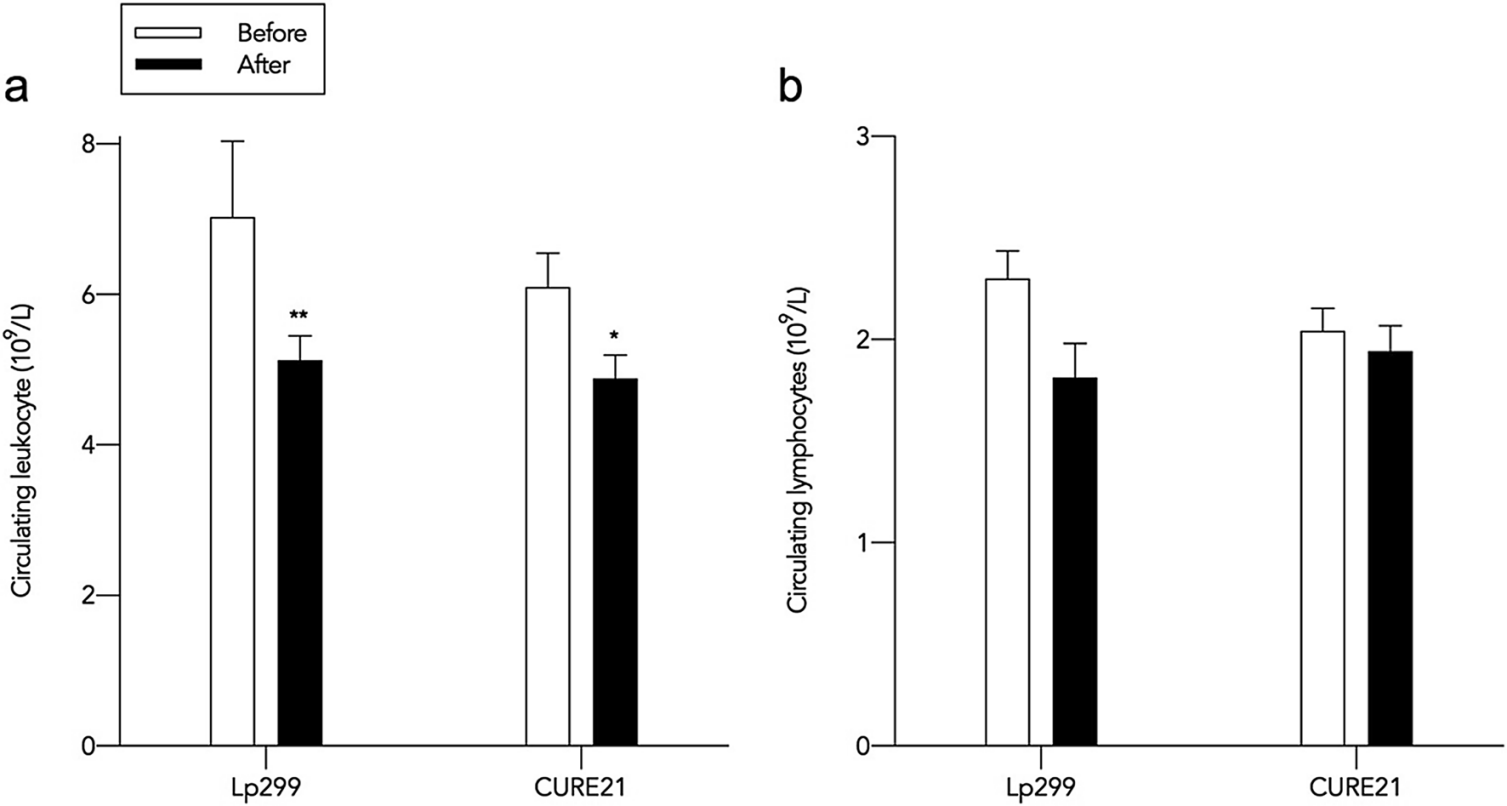
Total leukocyte (**a**) and lymphocyte (**b**) count. Levels are within normal range. Values are given as mean and SEM. *denotes *P* < 0.05 compared to before administration. **denotes *P* = 0.01 compared to before administration

Administration of Lp299 and CURE21 decreased the number of leukocytes but they stayed within normal range (Fig. 1a). The total concentration of lymphocytes has been normal during the study, with a decrease after intake of Lp299, however not significant (Fig. 1b).

CD3, CD4, CD8, CD19, CD16+56, HLA-DR, HLA-DR/CD3 and fibrinogen did not show any significant differences after ingestion of any of the probiotic strains while there was increasing expression of CD4 of CD25 after intake of both strains, although not significant (Table 2). The expression of CD3, CD4, CD8, CD19 and CD16+56 did not show any statistically significant difference after intake of either study product (Table 2). HLA-DR decreased but not significantly and HLA-DR/CD3 increased slightly after ingestion of both Lp299 and CURE21 (Table 2). CD4 of CD25 increased but not significantly after intake of both Lp299 and CURE21 (Table 2). Fibrinogen was normal during the study although we found a slight tendency to increase after administration of Lp299 and a slight decrease after CURE21 administration (Table 2). We did not see any changes in levels of CRP (all were <5 mg/L before as well as after administration of probiotics) or IL-6 in blood samples before and after administration of probiotics.

**Table 2:** FACS analysis

	Lp299		CURE21	
	Before	After	Before	After
Fibrinogen	2.54 ± 0.20	2.70 ± 0.20	3.11 ± 0.30	2.87 ± 0.22
CD3	1.76 ± 0.16	1.34 ± 0.14	1.51 ± 0.09	1.56 ± 0.15
CD4	1.08 ± 0.17	0.73 ± 0.06	0.95 ± 0.05	0.96 ± 0.11
CD8	0.56 ± 0.18	0.49 ± 0.12	0.51 ± 0.08	0.55 ± 0.09
CD19	0.20 ± 0.03	0.15 ± 0.03	0.23 ± 0.04	0.20 ± 0.03
CD16+56	0.31 ± 0.06	0.33 ± 0.05	0.26 ± 0.04	0.33 ± 0.04
HLR-DR	0.31 ± 0.06	0.35 ± 0.05	0.26 ± 0.04	0.33 ± 0.04
HLR-DR/CD3	0.07 ± 0.01	0.10 ± 0.03	0.08 ± 0.02	0.08 ± 0.02
CD25 of CD4	0.04 ± 0.01	0.05 ± 0.01	0.04 ± 0.00	0.05 ± 0.01

Data presented as mean ± SEM. Fibrinogen: g/L; CD, HLR-DR: x10^9^/L

### Gut microbiota

The presence of Lp299 could be verified in faecal samples from all persons ingesting the bacteria but CURE21 was only found in one out of seven individuals after ingestion.

The diversity of the intestinal microbiota, as assessed with T-RFLP, was not significantly different after the administration compared to before probiotic consumption for either Lp299 or CURE21 groups.

The median concentration of addressed bacterial taxa before and after probiotic consumption is depicted in Table 3. No statistically significant changes in the amount of the different taxa could be seen by the probiotic administration. No statistically significant difference could be seen between the subjects consuming *L. plantarum* 299 and those consuming *B. infantis* CURE21 when comparing bacterial load before and after probiotic ingestion.

**Table 3:** Concentrations of specific bacterial groups, detected by qPCR, in rectal tissue of healthy adults before and after 6 weeks probiotic consumption

Median (interquartile range)	Median (interquartile range)	
log 16 S rRNA copies/g tissue	log 16 S rRNA copies/g tissue	
Bbefore probiotic consumption	After probiotic consumption	*p* value
*Lactobacillus plantarum* 299		
(n = 6)		
*Lactobacillus*		
5.68 (5.68–5.94)	5.66 (5.65 –5.68)	0.180
*Bifidobacterium*		
6.33 (5.75–7.55)	6.75 (5.73–6.97)	0.589
*Akkermansia muciniphilia*-like		
6.74 (6.20–7.02)	6.55 (6.04–7.49)	1.000
*Bacteroides fragilis* group		
5.82 (5.82–6.77)	5.81 (5.71–5.96)	0.485
*Enterobacteriaceae*		
8.27 (8.06–10.01)	8.51 (7.73–9.46)	0.485
*Bifidobacterium infantis* CURE21		
(n = 4)		
*Lactobacillus*		
<5.65*	5.83 (5.65–6.41)	0.114
*Bifidobacterium*		
6.64 (6.12–7.48)	6.56 (5.57–7.14)	0.686
*Akkermansia muciniphilia*-like		
6.56 (5.98–7.28)	7.71 (6.62–8.21)	0.343
*Bacteroides fragilis* group		
5.86 (5.79–5.88)	5.92 (5.80–6.19)	0.343
*Enterobacteriaceae*		
7.73 (7.69–7.76)	8.15 (7.81–8.80)	0.114

Samples below detection limit were set to the detection limit of its specific qPCR assay

*All samples were below the detection limit of 5.65

### *In vitro* stimulation

LDH

The ratio of LDH in the supernatant over total activity was used to define viability and only samples with >60% viability were used for studies. All subjects had values <0.3 µkat/l, confirming that all tissue samples were viable.

IL-6

**Fig. 2:**
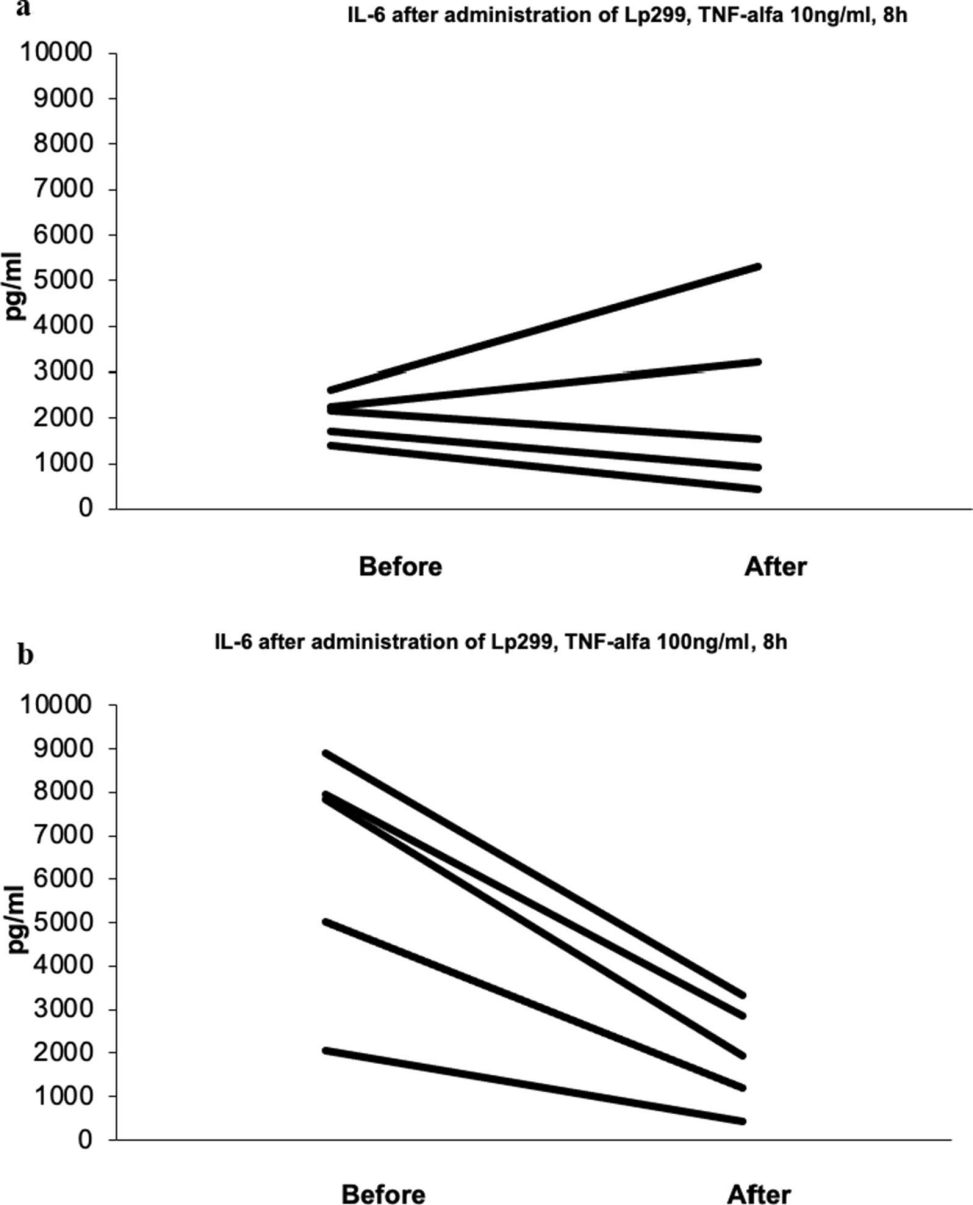
**a** Release of IL-6 after stimulation with TNF-α 10 ng/ml, 8 h, *in vitro*, Lp299-group. **b** Release of IL-6 after stimulation with TNF-α 100 ng/ml, 8 h, *in vitro*, Lp299-group

The administration of different bacteria evoked different responses to stimulation with TNF-α in release of IL-6. In the group with intake of Lp299 a reduced release of IL-6 was found upon stimulation with TNF-α 10 ng/ml. The reaction was more pronounced upon stimulation with the higher concentration of TNF-α 100 ng/ml as well as at a longer exposure and therefore only results for the latter period of 8 h are given (Fig. 2a, b). Two samples in the Lp299 group did not respond at all before start of ingestion of bacteria (IL-6 <200 pg/ml) and therefore excluded from analysis.

In the group with intake of CURE21 the response was more variable and there was no reactivity in three out of seven subjects whose samples were similarly excluded.

IL-10

There was no release of IL-10 at all time points or concentrations.

### Histology

**Fig. 3:**
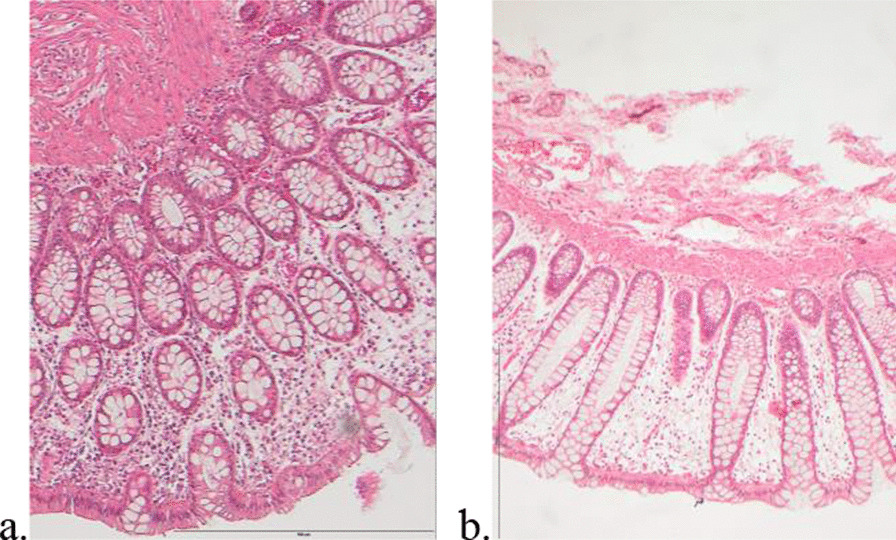
**a** and **b** Histology before and after administration of *L. plantarum* 299

In Haematoxylin-eosin staining we could observe a preserved architecture of the glands, Goblet cells and crypt structures in the colon before and after administration of probiotics as shown in Fig. 3a-b. No inflammation was observed.

## Discussion

In our study, the intake of *L. plantarum* 299 by healthy individuals is found to lead to a reduction of the pro-inflammatory cytokine IL-6 in rectal mucosa after *in vitro* stimulation with a high concentration of TNF-α. Ingestion of *B. infantis* CURE21 did not give the same response.

All volunteers completed the study without any complications. A subjective experience of improved gastrointestinal function was found, e.g. faecal consistency, gas production and abdominal pain, with better results for the CURE21 group. No other systemic effect was seen as was expected, as the persons were all without any present disease or infection.

Probiotics are widely used in different health products and are ascribed several functions upon ingestion, e.g. antibacterial, anti-inflammatory, anti-oxidative properties, to name a few. The strain Lp299 used in this study has previously shown its ability to colonize the gut mucosa upon ingestion. *L. plantarum* has been used by us and by many others in experimental and clinical studies and administration has been shown to reduce inflammation in patients under intensive care and with atherosclerosis [17, 22, 23].

For probiotics to have an effect on the gut mucosa we believe that the mucosa must adjust to an altered milieu after a long exposure and that the bacteria must colonize the mucosa. We thus chose to administer lactobacilli or bifidobacteria for 6 weeks.

*L. plantarum* could be found in biopsies from rectum in all subjects after ingestion of the study products, as has previously been shown by Johansson [20], showing its stability even at a distant site after oral ingestion. There were no alterations in the diversity of the dominating gut microbiota. This is in contrast to a study in men with atherosclerosis where administration of Lp 299v, another strain of *L. plantarum*, increased diversity [17]. Besides that, subjects in this study were without any previous diseases.

It is known that human gut microbiota shows high inter-individual variation, but the actual spatial distribution and co-occurrence patterns of gut mucosal microbiota seems to be important for maintaining a healthy intestinal mucosa [24].

In this study we investigated the effect of oral ingestion of two very different kind of probiotics. CURE21 could be retrieved in only one subject after six weeks. The reason for the poor recovery may be due to a low stability in an aerobic environment since the faeces was collected and stored in tubes for up to 24 h before analysis. CURE21 grows weakly on Rogosa agar and forms only tiny colonies possibly due to the high selective pressure of the substrate. The limited growth of the bifidobacteria could be expected since Lp299 can easily be re-isolated from faeces by plate count on Rogosa agar, whilst there is no equally good selective substrate for CURE21. The different behaviour of the probiotics in this experiment may be an explanation for the lack of effect.

The healthy volunteers were randomised to receive either lactobacilli or bifidobacteria, in order to elucidate if they share a common mechanism for their local or systemic anti-inflammatory effect. It should be hold in mind that lactobacilli and bifidobacteria, in a phylogenetical perspective, are very distant from each other as they belong to different phyla. It is important to study the effect of probiotic strains separately since the health effect can differ between strains. For example, recent meta-analyses have shown that there is strain-specific efficacy of probiotics in preventing gastrointestinal tract infections among infants and children attending childcare centres and in the treatment of irritable bowel syndrome [25, 26]. The scientific and clinically interesting question is if you can induce a strengthening of the mucosa before a potential insult, therefore we think that the relevant comparison is to compare before and after administration of the same probiotic bacterium.

The study was performed in healthy volunteers without any known disease and the only effect observed in the systemic circulation was a decrease in leukocyte counts reflecting a general state of decreased inflammation. Lymphocytes, fibrinogen and other inflammatory markers studied were found to be normal in the healthy individuals of the study.

An inflammatory injury is difficult to perform and standardize in subjects which is why we have chosen to perform the inflammatory exposure on mucosal biopsies *in vitro.* TNF-α exerts a pivotal role as a gut mucosal promotor of tissue degrading matrix metalloproteinases and proinflammatory cytokines e g IL-6 [27]. After ingestion of Lp299 decreases in the release of the pro-inflammatory cytokine IL-6 were observed after TNF-α-stimulation and the response seemed dose-dependent. There was no similar reduction in the release of IL-6 in biopsies from test persons after ingestion of CURE21. IL-10 showed no changes in any of the subjects and seems not to be affected by the simulated injury, suggesting another mechanism of anti-inflammatory way of action.

In this study we have tried to create a clinical condition with daily consumption of probiotics in order to allow the mucosa to adjust to different microbial ecology. We have then tried to standardize an inflammatory injury by exposing mucosal biopsies to TNF-α in increasing doses and at different time points. By this procedure we have in an *in vitro* system tried to simulate a standardized injury *in vivo* which otherwise is very difficult to do in a clinical setting. The results of the study indicate that the mucosa adapts well to probiotics and an inflammatory reaction is more pronounced in mucosa where probiotics are absent. There seems to be different reactivity in mucosal biopsies exposed to Lp299 and CURE21. The reason for this difference is uncertain but may reflect a different mode of interaction with the immune system by different bacteria. This difference of reactivity is not reflected systemically.

A limitation of this study is the low number of participants and the risk of placebo effect. We thus interpret our data with caution. However, to our knowledge, this is the first study of healthy mucosa exposed to a standardized inflammatory injury simulating radiation injury and analysed for the effects of probiotics. Since radiotherapy is increasingly used in the treatment of pelvic malignancies the burden of radiation induced injuries to the gut mucosa may be expected to increase in the future. Administration of probiotic bacteria may therefore be one way of preconditioning the mucosa to better withstand radiation injuries. A further strength of this study is the fact that the healthy volunteers constitute their own controls since they all are examined before as well as after ingestion of the two phylogenically different probiotic strains.

## Conclusions

Administration of the probiotic strains *Lactiplantibacillus plantarum *299 or *Bifidobacterium infantis* CURE21 to healthy humans show both systemic and local reduction of inflammatory response by lowering leukocyte counts, and for Lp 299 levels of IL-6 in rectal mucosa, after a standardized inflammatory stimulus *in vitro*. The results of the study may have an impact in several clinical situations where an injury to the intestinal mucosa might be anticipated and preferably action taken to avoid this. Situations where one may have to consider probiotic treatment or ingestion could be e.g. prior to major surgery, organ transplantation and before start of radio- or chemotherapy.

## Data Availability

The datasets used and/or analysed during this study are available from the corresponding author on reasonable request.

## Abbreviations

Lp299: *Lactiplantibacillus plantarum* 299
CURE21: *Bifidobacterium infantis* CURE21
CURE: Colonic ultima ratio efflux
LDH: Lactate dehydrogenase
CFU: Colony Forming Units
TNF-α: Tumour necrosis factor alpha
OFI: Oligofructose and Inulin
DSS: Dextran sulfate sodium
GIT: Gastrointestinal tract
DSM: Deutsche Sammlung von Mikroorganismen
T-RFLP: Terminal restriction fragment length polymorphism
PCR: Polymerase chain reaction
VRBG: Violet red bile glucose
qRAPD: Quantitative randomly amplified polymorphic DNA
CRP: C-reactive protein
FACS: Fluorescence activated cell sorting
CD: Cluster of differentiation
HLA-DR: Human leukocyte antigen-DR
IL-6: Interleukin 6
IL-10: Interleukin 10
DMEM: Dulbecco’s modified Eagle’s medium
PEST: Penicillin/Streptomycin
FCS: Fetal calf serum
ELISA: Enzyme-Linked ImmunoSorbent Assay
BMI: Body Mass Index
